# Leveraging functional annotations in genetic risk prediction for human complex diseases

**DOI:** 10.1371/journal.pcbi.1005589

**Published:** 2017-06-08

**Authors:** Yiming Hu, Qiongshi Lu, Ryan Powles, Xinwei Yao, Can Yang, Fang Fang, Xinran Xu, Hongyu Zhao

**Affiliations:** 1 Department of Biostatistics, Yale School of Public Health, New Haven, CT, United States of America; 2 Program of Computational Biology and Bioinformatics, Yale University, New Haven, CT, United States of America; 3 Yale College, New Haven, CT, United States of America; 4 Department of Mathematics, Hong Kong Baptist University, Kowloon, Hong Kong; 5 Department of Genetics, Yale University School of Medicine, New Haven, CT, United States of America; 6 Clinical Epidemiology Research Center (CERC), Veterans Affairs (VA) Cooperative Studies Program, VA Connecticut Healthcare System, West Haven, CT, United States of America; Thomas Jefferson University, UNITED STATES

## Abstract

Genetic risk prediction is an important goal in human genetics research and precision medicine. Accurate prediction models will have great impacts on both disease prevention and early treatment strategies. Despite the identification of thousands of disease-associated genetic variants through genome wide association studies (GWAS), genetic risk prediction accuracy remains moderate for most diseases, which is largely due to the challenges in both identifying all the functionally relevant variants and accurately estimating their effect sizes in the presence of linkage disequilibrium. In this paper, we introduce AnnoPred, a principled framework that leverages diverse types of genomic and epigenomic functional annotations in genetic risk prediction for complex diseases. AnnoPred is trained using GWAS summary statistics in a Bayesian framework in which we explicitly model various functional annotations and allow for linkage disequilibrium estimated from reference genotype data. Compared with state-of-the-art risk prediction methods, AnnoPred achieves consistently improved prediction accuracy in both extensive simulations and real data.

## Author summary

Genetic risk prediction plays a significant role in precision medicine. Accurate prediction models could have great impact on disease prevention and early treatment strategies. For example, mutations in *BRCA1* and *BRCA2* have been used to evaluate women’s breast cancer risk and as a guideline for early screening. However, genetic risk prediction models also present important challenges, including extreme high-dimensionality, limited access to and efficient computational methods for individual-level genotype data. To make use of rich GWAS summary statistics, we propose a novel method to address these challenges by integrating genomic functional annotations, which have been successfully applied in GWAS to generate biological insights. We demonstrate the improvement in accuracy in both extensive simulation studies and real data analysis of breast cancer, Crohn’s disease, celiac disease, rheumatoid arthritis and type-II diabetes.

This is a *PLOS Computational Biology* Methods paper.

## Introduction

Achieving accurate disease risk prediction using genetic information is a major goal in human genetics research and precision medicine. Accurate prediction models will have great impacts on disease prevention and early treatment strategies [1]. Advancements in high-throughput genotyping technologies and imputation techniques have greatly accelerated discoveries in genome-wide association studies (GWAS) [2]. Various approaches that utilize genome-wide data in genetic risk prediction have been proposed, including machine-learning models trained on individual-level genotype and phenotype data [3–8], and polygenic risk scores (PRS) estimated using GWAS summary statistics [9, 10]. Despite the potential information loss in summary data, PRS-based approaches have been widely adopted in practice since the summary statistics for large-scale association studies are often easily accessible [11, 12] while individual-level data are more difficult to acquire, deposit, and process. However, prediction accuracies for most complex diseases remain moderate, which is largely due to the challenges in both identifying all the functionally relevant variants and accurately estimating their effect sizes in the presence of linkage disequilibrium (LD) [13].

Explicit modeling and incorporation of external information, e.g. pleiotropy [7, 8] and LD [10], has been shown to effectively improve risk prediction accuracy. Recent advancements in integrative genomic functional annotation, coupled with the rich collection of summary statistics from GWAS, have enabled increase of statistical power in several different settings [14–16]. To our knowledge, the impact of functional annotations on performance of genetic risk prediction has not been systematically studied. Here, we introduce AnnoPred (available at https://github.com/yiminghu/AnnoPred), a principled framework that integrates GWAS summary statistics with various types of annotation data to improve risk prediction accuracy. We compare AnnoPred with state-of-the-art PRS-based approaches and demonstrate its consistent improvement in risk prediction performance using both simulations and real data of multiple complex diseases.

AnnoPred risk prediction framework has three main stages (**Methods**). First, we estimate GWAS signal enrichment in 61 different annotation categories, including functional genome predicted by GenoCanyon scores [17], GenoSkyline tissue-specific functionality scores of 7 tissue types [14], and 53 baseline annotations for diverse genomic features [18] for each trait analyzed. Second, we propose an empirical prior of SNP effect size based on annotation assignment and signal enrichment. In general, SNPs located in annotation categories that are highly enriched for GWAS signals receive a higher effect size prior. Finally, the empirical prior is adopted in a Bayesian framework in which marginal summary statistics and LD matrix estimated from a reference panel are jointly modeled to infer the posterior effect size of each SNP. AnnoPred PRS is defined by

$$PRS=\sum_{j=1}^{M}X_{j}E_{A}(\beta_{j}|\hat{\beta },\hat{D})$$

where *X*_*j*_ and *β*_*j*_ are the standardized genotype and effect size of the j^th^ SNP, respectively, $\hat{\beta }$ is the marginal estimate of *β*, $\hat{D}$ is the sample LD matrix, and $E_{A}(\beta_{j}|\hat{\beta },\hat{D})$ denotes the posterior expectation of effect sizes under an empirical prior based on annotation assignment for all SNPs when adjusting for LD matrix estimated from a reference panel (**Methods**).

## Results

**Fig 1:**
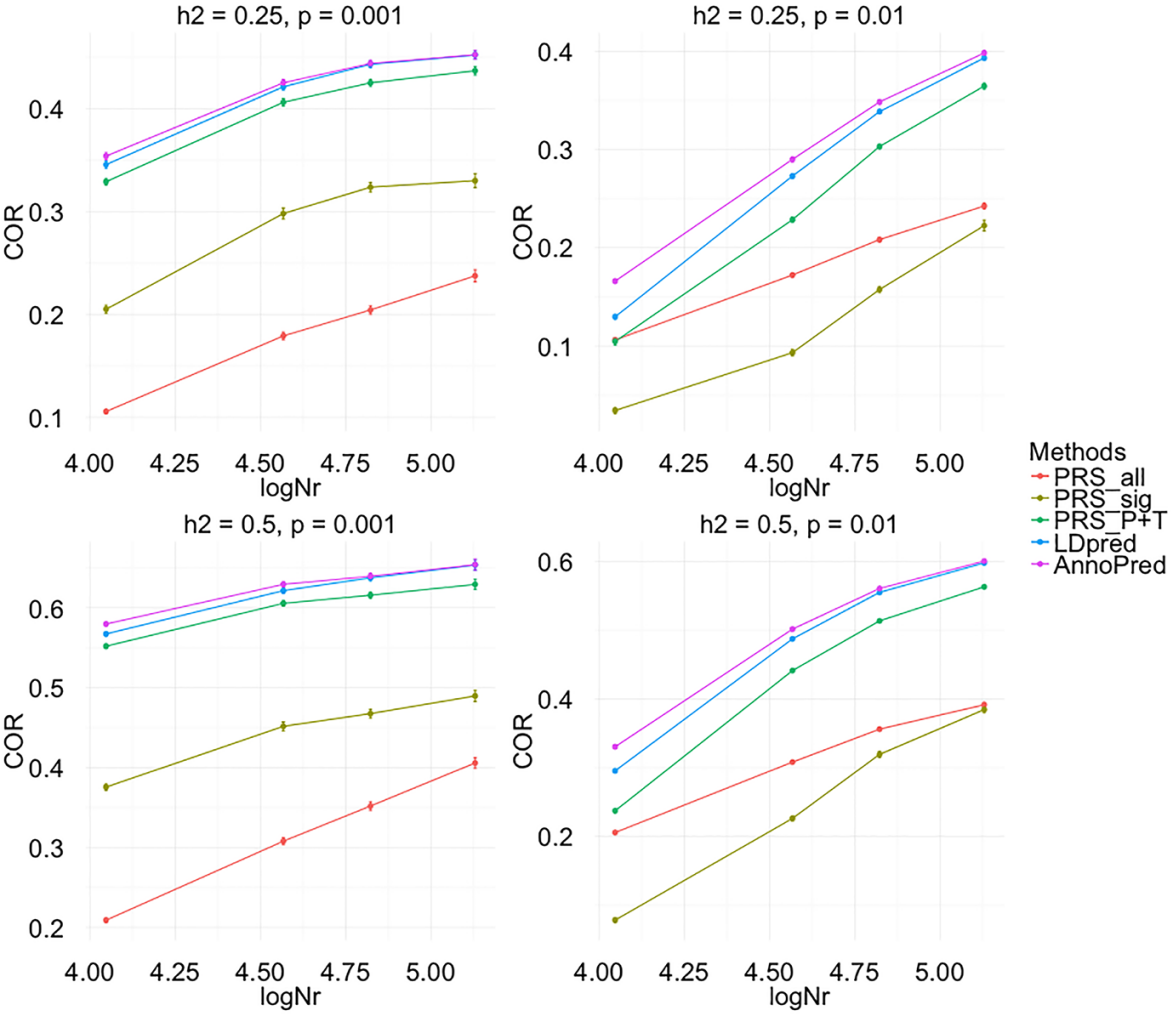
Evaluating the effect of sample size on prediction accuracy in simulation. Traits were simulated using SNPs of chromosome 1, chromosome 1 and 2, chromosome 1 to 4 and the whole genome while keeping the same proportion of causal variants and heritability to mimic the situation of increasing sample size. In the figure, logNr = $\log N\frac{M}{M_{s}}$, where *N* is the number of individuals, *M* is the total number of variants and *M*_*s*_ is the number of variants used in simulation. In total four settings were simulated for each effective sample size: *h*^2^ = 0.25, *p* = 0.001; *h*^2^ = 0.25, *p* = 0.01; *h*^2^ = 0.5, *p* = 0.001; *h*^2^ = 0.5, *p* = 0.01, where *p* represents the proportion of causal variants. Each dot represent the mean COR of 50 replicates in one simulation setting and error bar represents the standard error.

We first performed simulations to demonstrate AnnoPred’s ability to improve risk prediction accuracy. We compared AnnoPred with four popular PRS approaches (**Methods**), including PRS based on genome-wide significant SNPs (PRS_sig_), PRS based on all SNPs in the dataset (PRS_all_), PRS based on tuned cutoffs for p-values and LD pruning (PRS_P+T_), and recently proposed LDpred [10]. Mean correlations between simulated and predicted traits were calculated from 100 replicates under different simulation settings (**Methods**). AnnoPred showed the best prediction performance in all settings when the causal SNPs are highly enriched in annotated regions (**Table 1, S2 Table and S2 Fig**). In general, performance of PRS_sig_, PRS_P+T_, LDpred, and AnnoPred all improved under a sparser genetic model and higher trait heritability. PRS_all_ showed comparable performance between sparse and polygenic models but its prediction accuracy was consistently worse than other methods. Sample size in the training set was also crucial for risk prediction accuracy. Increasing sample size could lead to continuous improvement in prediction accuracy under different settings (**Fig 1**).

**Table 1:** Mean correlation between simulated and predicted traits calculated from 100 replicates under different simulation settings. The highest mean correlations are highlighted in boldface. Standard deviations are shown in parentheses. Traits were simulated from WTCCC genotype data, which contain 15,918 individuals genotyped for 393,273 SNPs. In each setting, we used 70% of the data to calculate the training summary statistics and randomly divided the rest 30% into two parts for parameter tuning.

Training samples	Heritability	#Causal	PRS_sig_	PRS_all_	PRS_P+T_	LDpred	AnnoPred
Half (~5K)	0.25	300	0.149(.028)	0.08(.021)	0.25(.028)	0.279(.025)	**0.286**(.024)
		3000	NA*	0.082(.016)	0.073(.020)	0.087(.019)	**0.096**(.020)
	0.5	300	0.304(.04)	0.16(.022)	0.48(.026)	0.502(.033)	**0.512**(.026)
		3000	NA*	0.157(.019)	0.157(.024)	0.195(.021)	**0.209**(.019)
Full (~10K)	0.25	300	0.217(.031)	0.11(.02)	0.332(.023)	0.35(.033)	**0.358**(.022)
		3000	NA*	0.11(.014)	0.107(.018)	0.136(.017)	**0.145**(.017)
	0.5	300	0.373(.036)	0.213(.023)	0.548(.024)	0.557(.047)	**0.566**(.034)
		3000	0.078(.023)	0.21(.019)	0.243(.021)	0.309(.021)	**0.324**(.019)

* NA means no SNP achieves genome-wide significance level (5e-8).

**Fig 2:**
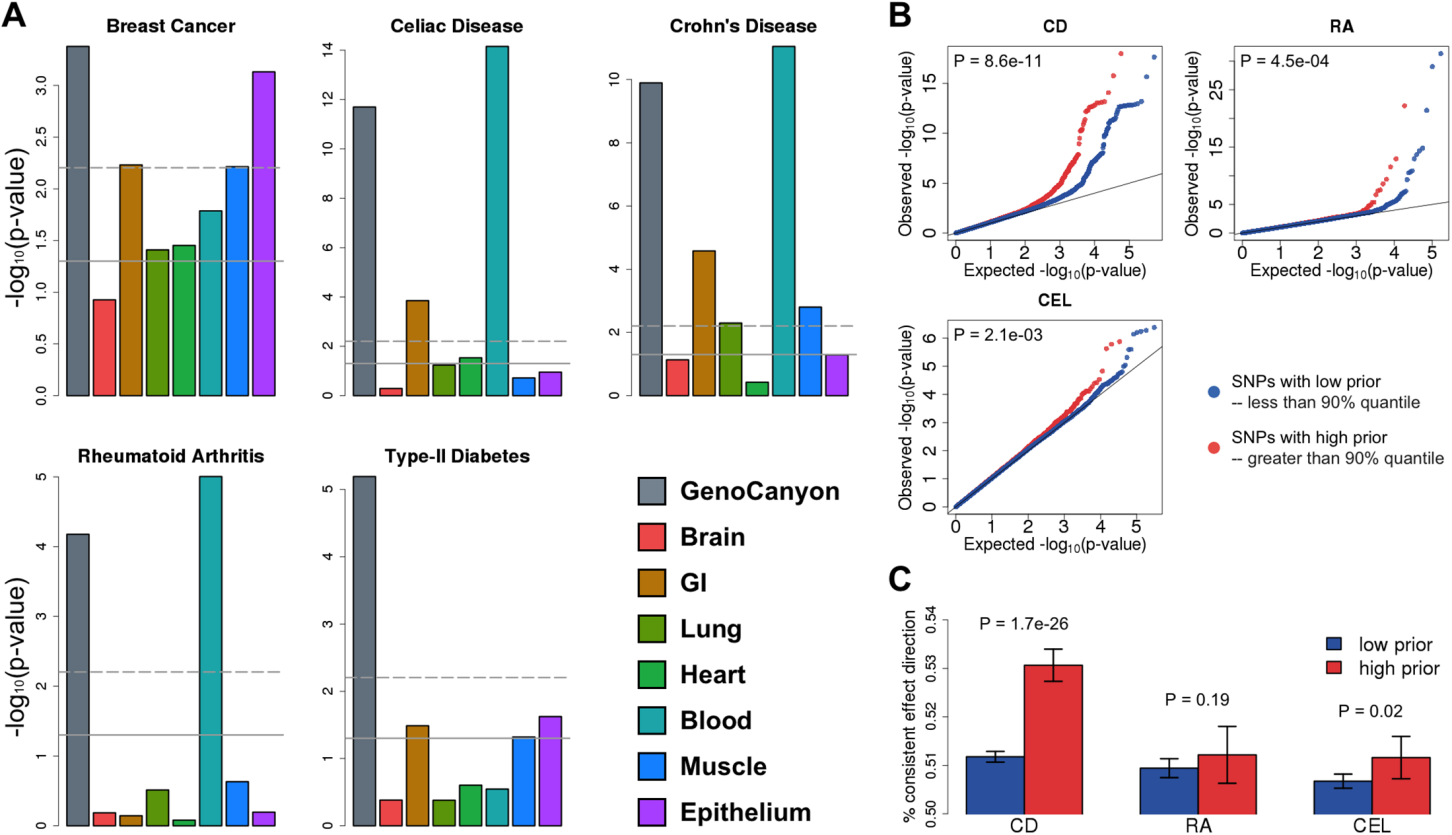
Evaluating effectiveness of annotations and empirical effect size prior. **(A)** GWAS signal enrichment across GenoCanyon and tissue-specific GenoSkyline annotations. The horizontal lines mark p-value cutoffs of 0.05 and Bonferroni corrected significance level. **(B)** Comparing signal strength of SNPs with high priors and low priors in independent validation cohorts. SNPs with higher priors have significantly stronger associations across three independent and well-powered testing datasets (N>2,000). P-values were calculated using one-sided Kolmogorov-Smirnov test. **(C)** Comparing consistency of SNPs’ effect direction between training and testing datasets. Each bar quantifies the proportion of SNPs with consistent effect directions. P-values were calculated using one-sided two-sample binomial test.

To illustrate the improved risk prediction performance in real data, we applied AnnoPred to five human complex diseases—Crohn’s disease (CD), breast cancer (BC), rheumatoid arthritis (RA), type-II diabetes (T2D), and celiac disease (CEL). We first estimated GWAS signal enrichment in different annotation categories (**Methods**). Enrichment pattern varies greatly across diseases (**Fig 2A**; **S1 Table**), reflecting the genetic basis of these complex phenotypes. Functional genome predicted by GenoCanyon was consistently and significantly enriched for all five diseases. Blood was strongly enriched for three immune diseases, namely CD (P = 8.9×10^−12^), CEL (P = 7.0×10^−15^), and RA (P = 9.9×10^−6^), while gastrointestinal (GI) tract was enriched in CD (P = 2.6×10^−5^) and CEL (P = 1.4×10^−4^), both of which have a known GI component. For BC, epithelium (P = 7.4×10^−4^), GI (P = 5.9×10^−3^), and muscle (P = 6.1×10^−3^) were significantly enriched. A few studies have shown that breast cancer could arise from epithelial cells [19, 20]. The connections between breast cancer and muscle as well as GI tract have also been previously suggested [21, 22]. In addition, studies have suggested that GI can be used as diagnostic and treatment target for type-II diabetes, Crohn’s disease, and celiac disease [23–25]. Furthermore, the connection between immune system and Crohn’s disease, celiac disease and rheumatoid arthritis have been extensively studied in literature [26–28]. Next, we evaluated the effectiveness of proposed empirical effect size prior in three diseases (i.e. CD, CEL, and RA) with well-powered testing cohorts (N>2,000). Interestingly, despite the highly variable enrichment results in training datasets, integrative effect size prior could effectively identify SNPs with large effect sizes and consistent effect directions in independent validation cohorts (**Fig 2B and 2C**).

Correlations between the calculated PRS and disease status (COR) for different approaches are summarized in **Table 2**. AnnoPred showed consistently improved prediction accuracy compared with all other methods across five diseases. Notably, PRS_sig_ and PRS_all_ showed suboptimal performance in these datasets, reaffirming the importance of modeling LD and other external information. A likelihood ratio test was used to test for the difference in the prediction accuracy between models comparing the likelihood of a logistic regression fitting PRS of one method to that of a logistic regression fitting PRS of two methods jointly (**S11 Table**). From the test, AnnoPred with 61 annotations performed significantly better than LDpred (p = 1.2E-22 for CD, p = 0.045 for BC, p = 4.2E-7 for RA, p = 3.3E-4 for T2D and p = 1.3E-3 for CEL). Reversing the order of test (that is, comparing the likelihood of model using annotations with model using and not using annotations jointly) results in non-significant p-values for most tests (**S11 Table**), which further demonstrates that PRS incorporating functional annotations mostly encompasses the information of PRS without annotations. To test different methods’ ability to stratify individuals with high risk, we compared the proportion of cases among testing samples with high PRS. AnnoPred outperformed all other methods in CD, CEL, RA, and T2D (**S1 Fig**). Next, we tested AnnoPred’s performance using only the 53 baseline annotations and observed a substantial drop in prediction accuracy for all diseases (**S3 Table**). AnnoPred with GenoCanyon and GenoSkyline annotations only (nine annotation tracks in total) yields better performance than the 53 baseline annotations (**S10 Table**). For CD and T2D, by using these 9 categories AnnoPred even achieved higher accuracy than the model with all 61 annotation tracks added. These results highlight the importance of annotation quality in genetic risk prediction, and also demonstrate GenoCanyon and GenoSkyline’s ability to accurately identify functionality in the human genome. Since different diseases have various enrichment patterns, we also run AnnoPred with significantly enriched annotations (enrichment test p value less than 0.05) for each disease (**S10 Table**). In general, using only the significantly enriched annotations indeed improved the performance in most diseases.

**Table 2:** CORs of different methods. The highest CORs are highlighted in boldface.

Disease/Trait	PRS_sig_	PRS_all_	PRS_P+T_	LDpred	AnnoPred
Crohn's Disease	0.27	0.229	0.32	0.325	**0.343**
Breast Cancer	0.084	0.055	0.12	0.122	**0.137**
Rheumatoid Arthritis	0.204	0.114	0.248	0.282	**0.287**
Type-II Diabetes	0.165	0.156	0.204	0.202	**0.22**
Celiac Disease	0.11	0.136	0.18	0.197	**0.213**

Tissue specificity plays an important role in genetic risk prediction. Integrating more functional annotations with higher tissue and cell type specificity may further increase risk prediction accuracy, especially when the tissue type that is biologically relevant to the disease is not well characterized by the seven available tissue tracks in our current analyses. To explore how these factors will affect the AnnoPred model, we performed a few follow-up analyses. We have recently expanded our GenoSkyline annotations to more than 100 tissue and cell types from the Roadmap Epigenomics Project [29]. We investigated the performance of AnnoPred after integrating 66 annotation tracks representing a spectrum of adult tissue and cell types. As shown in S10 Table, incorporating more annotations into the model does not always further improve risk prediction accuracy compared with AnnoPred with fewer annotations in the model. This may be due to the overlap between functional regions (e.g. functional annotations for slightly different brain regions) when incorporating too many annotation tracks into the model, which will cause numerically unstable heritability estimates. This is because annotation-stratified LD score regression, the method we used to empirically estimate the informative prior for SNPs’ effect sizes, is a multiple linear regression model that regresses SNP-level summary statistics against annotation-stratified LD scores. When two functional annotation tracks are similar, the corresponding LD scores will also be correlated by definition. It is well understood that if multi-collinearity (i.e. correlation among covariates) in multiple regression leads to numerically unstable estimates for regression coefficients [30] (the heritability parameters in our case).

In order to study the effect of highly associated SNPs (e.g. SNPs in MHC regions for immune traits), we repeated the analysis on CD, RA, BC and T2D after removing the SNPs in MHC region (chr6: 28,477,797–33,448,354 bp). Re-analysis of CEL was unnecessary since the training summary statistics of CEL does not contain any SNP in the MHC region. After removing SNPs in MHC regions, the prediction accuracies for RA drops dramatically for all methods and AnnoPred remained to be the method with the best performance (**S9 Table**). For the rest diseases, results varied little from the original analysis. Besides COR, we also included AUCs for all the analysis performed (**S2, S6, S9** and **S10 Tables**), all of which showed consistent patterns.

Due to distinct allele frequencies and LD structures across populations, risk prediction accuracy usually drops when the training and testing samples are from different populations. In order to investigate the robustness of AnnoPred against population heterogeneity, we applied AnnoPred to three non-European cohorts for breast cancer and type-II diabetes while training the model using summary statistics from European-based studies. The CORs and AUCs are summarized in **S6 and S7 Tables**. As expected, we observed a drop in prediction accuracy for all methods. However, AnnoPred still performed the best in all three trans-ethnic validation datasets.

## Discussion

Our work demonstrates that functional annotations can effectively improve performance of genetic risk prediction. AnnoPred jointly analyzes diverse types of annotation data and GWAS summary statistics to upweight SNPs with a higher likelihood of functionality, which lead to consistently better prediction accuracy for multiple complex diseases. Our method is not without limitation. First, despite the consistent improvement compared with existing PRS-based methods, accuracies for most diseases remain moderate. In order to effectively stratify risk groups for clinical usage, our model remains to be further calibrated using large cohorts with measured environmental and clinical risk factors [1]. Second, accurate estimation of GWAS signal enrichment and SNP effect sizes requires a large sample size for the training dataset. This could potentially be improved by new estimators for annotation-stratified heritability [19]. A few Bayesian models combining GWAS summary statistics with functional annotations have been proposed for the purpose of fine-mapping functional variants [16, 20, 21]. Whether these models could be adapted to benefit risk prediction accuracy remains to be investigated in the future. Importantly, the rich collection of publicly available integrative annotation data, in conjunction with the increasing accessibility of GWAS summary statistics, makes AnnoPred a customizable and powerful tool. As GWAS sample size continues to grow, AnnoPred has the potential to achieve even better prediction accuracy and become widely adopted as a summary of genetic contribution in clinical applications of risk prediction.

## Methods

### Annotation data

GenoCanyon is a statistical framework to predict functional regions in the human genome through integrative analysis of ENCODE epigenomic data and multiple conservation metrics [17]. Later we have further extended the model and developed GenoSkyline, which aimed to predict tissue-specific functionality [14]. In the AnnoPred model, we incorporated GenoCanyon general functionality scores, GenoSkyline tissue-specific functionality scores for seven tissue types (brain, gastrointestinal tract, lung, heart, blood, muscle, and epithelium), and 53 LDSC baseline annotations that covered a variety of genomic features [18] (**S1 Table**). We smoothed GenoCanyon scores by a 10Kb window, a strategy previously shown to improve robustness of functionality prediction [22]. The smoothed GenoCanyon annotation and raw GenoSkyline annotations of seven tissue types were dichotomized based on a cutoff of 0.5. The regions with GenoCanyon or GenoSkyline scores greater than the cutoff were interpreted as non-tissue-specific or tissue-specific functional regions in the human genome. Such dichotomization has been previously shown to be robust against the cutoff choice [14]. Notably, the AnnoPred framework allows users to specify their own choice of annotations.

### Heritability partition

We assume throughout the paper that both the phenotype *Y*_*N*×1_ and the genotypes *X*_*N*×*M*_ are standardized with mean zero and variance one. We assume a linear model

$$Y_{N\times 1}=X_{N\times M}\beta_{M\times 1}+\varepsilon_{N\times 1}$$

*X*, *β* and *ε* are mutually independent. We also assume that *β* is a random effect and effects of different SNPs are independent. A key idea in the AnnoPred framework is to utilize functional annotation information to accurately estimate SNPs’ effect sizes. In order to achieve that, we first partition trait heritability by annotations using LD score regression [18]. Since genotypes are standardized, per-SNP heritability is defined as the variance of *β*_*i*_ for the i^th^ SNP, and is used to quantify SNP effect sizes. More specifically, assume there are *K* + 1 pre-defined annotation categories, denoted as *S*_0_, *S*_1_, …, *S*_*K*_ with *S*_0_ representing the entire genome. Under an additive assumption for heritability in overlapped annotations, we have $\beta_{i}∼N(0,\sum_{j:i\in S_{j}}\tau_{j})$, where *τ*_0_, *τ*_1_, …, *τ*_*K*_, quantify the contribution to per-SNP heritability from each annotation category. Denote the estimated marginal effect size of the i^th^ SNP as ${\hat{\beta }}_{i}=\frac{X_{i}^{T}Y}{N}$, then we have the following approximation

$$E(N{\hat{\beta }}_{i}^{2})\approx (N-1)\sum_{k}\tau_{k}l(i,k)+1$$

where *l*(*i*, *k*) is the annotation-stratified LD score and *N* denotes the total sample size. Regression coefficients *τ*_*k*_ are estimated through weighted least squares. The estimated heritability of the i^th^ SNP is then $\hat{Var}(\beta_{i})=\sum_{j:i\in S_{j}}{\hat{\tau }}_{j}$.

### Empirical prior of effect size

Based on per-SNP heritability estimates, we propose two different priors for SNP effect sizes to add flexibility against different genetic architecture. For the first prior, we assume that SNP effect size follows a spike-and-slab distribution

βi∼p0N(0,σ^i2p0)+(1−p0)δ0

where *p*_0_ is the proportion of causal SNPs in the dataset, and *δ*_0_ is a Dirac function representing a point mass at zero. The empirical variance of each SNP, i.e. ${\hat{\sigma }}_{i}^{2}$, is determined by the annotation categories it falls in. More specifically, we assume ${\hat{\sigma }}_{i}^{2}=c(\sum_{j:i\in S_{j}}{\hat{\tau }}_{j})$, where *c* is a constant calculated from the following equation

$$\sum_{i}{\hat{\sigma }}_{i}^{2}={\hat{H}}^{2}.$$

We do not directly use $\sum_{j:i\in S_{j}}{\hat{\tau }}_{j}$ as the empirical variance prior because it is estimated in the context where all SNPs in the 1000 Genomes Project database are included in the model [18]. Such per-SNP heritability estimates cannot be extrapolated to the risk prediction context where many fewer SNPs are analyzed [23]. Therefore, we rescale the heritability estimates to better quantify each SNP’s contribution toward chip heritability. Following [24], we use a summary statistics-based heritability estimator that approximates the Haseman-Elston estimator:

$${\hat{H}}^{2}=\frac{\left({\bar{\chi }}^{2}-1\right)}{N\overset{-}{l}}$$

where ${\bar{\chi }}^{2}$ and $\bar{l}$ denote the mean $N{\hat{\beta }}_{i}^{2}$ and mean non-stratified LD score, respectively.

In the first prior, we assumed the same proportion of causal SNPs but different effect sizes across annotation categories. We now describe the second prior that assumes different proportions of causal SNPs but the same effect size across annotation categories. To be specific, we assume the causal effect size to be *Var*(*β*_*causal*_) = *V*, the total number of SNPs to be *M*_0,_ and the overall proportion of causal SNPs to be *p*_0._ The total heritability $H_{0}^{2}$ can then be written as $H_{0}^{2}=p_{0}M_{0}V$. For the i^th^ SNP, use $T_{i}=(\underset{j:i\in S_{j}}{⋂}S_{j})\cap (\underset{k:i\notin S_{k}}{⋂}S_{k}^{c})$ to denote the collection of SNPs that share the same annotation assignment with the i^th^ SNP, and let $M_{T_{i}}=|T_{i}|$, i.e. the number of SNPs in the set. Then, the total heritability of SNPs in *T*_*i*_ is $H_{T_{i}}^{2}=p_{T_{i}}M_{T_{i}}V$, with $p_{T_{i}}$ denoting the proportion of causal SNPs in *T*_*i*_. Following these notations, we have

$$\beta_{i}∼p_{T_{i}}N(0,V)+(1-p_{T_{i}})\delta_{0}$$

where $V=\frac{H_{0}}{p_{0}N_{0}}$ and $p_{T_{i}}=p_{0}\frac{M_{0}H_{T_{i}}^{2}}{M_{T_{i}}H_{0}^{2}}$. We use ${\hat{H}}^{2}$ to estimate $H_{0}^{2}$, and the following formula to estimate $H_{T_{i}}^{2}$.

$${\hat{H}}_{T_{i}}^{2}=\frac{\sum_{k\in T_{i}}\sum_{j:k\in S_{j}}{\hat{\tau }}_{j}}{\sum_{k=1}^{M_{0}}\sum_{j:k\in S_{j}}{\hat{\tau }}_{j}}{\hat{H}}^{2}$$

Finally, *p*_0_ is treated as a tuning parameter for both prior functions in our analysis.

### Calculation of posterior effect sizes

By Bayes’ rule, the posterior distribution of *β* is:

$$f(\beta |\hat{\beta },\hat{D})\propto f(\hat{\beta }|\beta ,\hat{D})f(\beta )$$

where $\hat{D}=\frac{1}{N}X^{T}X$ is the sample correlation matrix and $\hat{\beta }=\frac{1}{N}X^{T}Y$ is the marginal effect size estimates. Given *β* and $\hat{D}$, $\hat{\beta }$ follows a multivariate normal distribution asymptotically with the following mean and variance

$$E(\hat{\beta }|\beta ,\hat{D})=\frac{1}{N}[E(X^{T}X\beta |\beta ,\hat{D})+E(X^{T}\varepsilon |\beta ,\hat{D})]=\hat{D}\beta$$

$$Var(\hat{\beta }|\beta ,\hat{D})=Var(\frac{1}{N}X^{T}\varepsilon |\beta ,\hat{D})=\frac{1}{N}(1-h_{g}^{2})\hat{D}.$$

However, $\hat{D}$ is usually non-invertible and has very high dimensions. We thus study the posterior distribution of a small chunk of $\hat{\beta }$ instead. Let ${\hat{\beta }}_{b}$ be the estimated marginal effect size of SNPs in a region *b* (e.g. a LD block) and the corresponding genotype matrix is *X*_*b*_ and sample correlation matrix is ${\hat{D}}_{b}$. Then the conditional mean and variance of ${\hat{\beta }}_{b}$ are

$$E({\hat{\beta }}_{b}|\beta_{b},{\hat{D}}_{b})=\frac{1}{N}[E(X_{b}^{T}X\beta |\beta_{b},{\hat{D}}_{b})+E(X_{b}^{T}\varepsilon |\beta_{b},{\hat{D}}_{b})]={\hat{D}}_{b}\beta_{b}$$

$$\begin{array}{c} Var\left({\hat{\beta }}_{b}\text{|}\beta_{b},{\hat{D}}_{b}\right)=\frac{1}{N^{2}}var\left(X_{b}^{T}X_{b}\beta_{b}+X_{b}^{T}\left(X_{-b}\beta_{-b}+\varepsilon \right)|\beta_{b},{\hat{D}}_{b}\right)\\ =\frac{1}{N^{2}}var\left(X_{b}^{T}\left(X_{-b}\beta_{-b}+\varepsilon \right)|\beta_{b},{\hat{D}}_{b}\right)\\ =\frac{1}{N^{2}}X_{b}^{T}var\left(X_{-b}\beta_{-b}+\varepsilon \text{|}\beta_{b},{\hat{D}}_{b}\right)X_{b}\\ =\frac{1}{N}\left(1-h_{b}^{2}\right){\hat{D}}_{b} \end{array}$$

where $h_{b}^{2}=\sum_{i\in b}\sigma_{i}^{2}$ is the heritability of SNPs in region *b*, and *X*_−*b*_ and *β*_−*b*_ denote the genotype matrix and effect sizes of SNPs not in region *b*. The conditional distribution of *β*_*b*_ is:

f(βb|β^b,D^b)∝N(D^bβb,1N(1−hb2)D^b)∏i∈bf(βi)∝{N(D^bβb,1N(1−hb2)D^b)∏i∈b[p0N(0,σi2p0)+(1−p0)δ0],underthefirstpriorN(D^bβb,1N(1−hb2)D^b)∏i∈b[pTiN(0,V)+(1−pTi)δ0],underthesecondprior

Although it is difficult to derive $E(\beta_{b}|{\hat{\beta }}_{b},{\hat{D}}_{b})$ from the joint conditional distribution of *β*_*b*_, each element of *β*_*b*_ follows a mixed normal distribution conditioning on ${\hat{\beta }}_{b}$, ${\hat{D}}_{b}$, and all other elements in *β*_*b*_. Therefore, we apply a Gibbs sampler to draw samples from $f(\beta_{b}|{\hat{\beta }}_{b},{\hat{D}}_{b})$ and use the sample mean as an approximation for $E(\beta_{b}|{\hat{\beta }}_{b},{\hat{D}}_{b})$. We further performed a sensitivity analysis on the choice of the size of block *b* (**S6 Fig**). Specifically, we ran AnnoPred on the data of Crohn’s disease with different sizes of block and found that the results were robust to the sizes. In practice, the size of block *b* is specified by the total number of variants divided by 3,000.

### Calculation of PRS

PRS is calculated using the following formula

$$PRS=\sum_{j=1}^{M}X_{j}E_{A}(\beta_{j}|\hat{\beta },\hat{D}),$$

where *E*_*A*_ denotes the posterior expectation as described above. In practice, the individual-level genotype matrix is not available and we use the LD matrix estimated from a reference panel or the validation samples to substitute $\hat{D}$. We apply the same standard of choosing the size of *b* as described in [10]. Choices of prior and *p*_0_ can be tuned in an independent cohort. For the data analysis described in this work, we adopted a cross-validation scheme to select tuning parameter due to the challenge in finding multiple independent cohorts without overlapping with the training GWAS summary statistics. The training datasets in our real data analyses and simulations are always fixed, i.e. GWAS summary statistics. We did not perform a classical cross-validation by using different subsets of the complete data to train and test our prediction model. The purpose of cross-validation in our study is purely parameter tuning. To select a suitable tuning parameter, we divide the independent testing dataset (individual level genotype and phenotype data) into two equal parts (A and B), and select the tuning parameters by optimizing prediction accuracy on dataset A. We then evaluate prediction accuracy using the remaining half of testing data, i.e. dataset B. Finally, we repeat the analysis one more time by choosing the tuning parameter on dataset B while evaluating the prediction accuracy on dataset A. Results from these two separate analyses are averaged to quantify model performance. For T2D where multiple independent cohorts are available (phs000237 and phs000388), we used an independent cohort for parameter tuning and the other for evaluating performance (**S12 Table**). The results are consistent with the cross-validation.

### Comparison with existing methods

We compared AnnoPred with several commonly used risk prediction methods based on summary data of association studies. PRS_sig_ and PRS_all_ were both calculated as the inner product of marginal effect size estimates and the corresponding genotypes. PRS_all_ used all the SNPs that are shared between training and testing datasets while PRS_sig_ only used SNPs with p-values below 5 × 10^−8^ in the training set. PRS_P+T_ used SNPs passing both LD pruning and p-value thresholding. The thresholds are tuned in an independent dataset over a grid (0, 0.1, 0.2, … 0.9 for LD; 1, 0.3, 0.1, 0.03, 0.01, 3E-3, 1E-3, 3E-4, 1E-4, 3E-5, 1E-5, 1E-6, 1E-7, 5E-8, 1E-8 for p-value). LDpred can be viewed as a special case of AnnoPred, assuming the whole genome as the only functional annotation. This is because when enrichment is constant (i.e. causal variants are uniformly distributed across the genome), per-SNP heritability estimates would be nearly constant and therefore results in similar performance to LDpred. We have performed an additional simulation to demonstrate this using WTCCC genotype data with ~15K individuals and ~330K variants. We randomly divided the genome into two parts (two annotations) and uniformly selected causal SNPs. Then the traits were simulated in a similar way as other simulations in this paper. We estimated per-SNP heritability using LDSC in the two annotation categories, respectively. We ran the procedure for 100 times and the distributions of estimated per-SNP heritability in both regions are summarized in the figure below (the dashed line denotes the true per-SNP heritability, added as S4 Fig in the manuscript), which indicates that the per-SNP heritability estimates are uniform across the genome under constant enrichment. Therefore, AnnoPred would be mathematically equivalent with LDpred with enrichment is constant. We downloaded python code for PRS_P+T_ and LDpred from Github (https://github.com/bvilhjal/ldpred). All the tuning parameters were tuned through cross-validation as we did for AnnoPred. Besides all these PRSs, we also compared AnnoPred with a evaluating method used in [5], which uses 1E-1, 1E-2,…, 1E-5 as p-value threshold to select SNPs and report the accuracy for the best performed threshold (**S4 and S5 Tables**).

Given that many large-scale GWAS summary statistics have included almost all available cohorts for a disease of interest, it is challenging to find independent datasets with individual-level genotype and phenotype information and sufficient sample sizes. We were able to identify ideal validation datasets for the five diseases we analyzed in this paper. The performance of different methods on more traits shall be evaluated when we get access to more data in the future.

### Simulation settings

We simulated traits from WTCCC genotype data, which contain 15,918 individuals genotyped for 393,273 SNPs after filtering variants with missing rate above 1% and individuals with genetic relatedness above 0.05. We first generated two annotations and each annotation was simulated by randomly selecting 10% of the genome, denoted as *A*_1_ and *A*_2_, which we assume are known when applying AnnoPred. Denote the heritability of the trait as $h_{g}^{2}$ (25% or 50%) and the number of causal variants as *m* (300 or 3,000). Causal variants were generated as follows: m3 causal variants were selected from *A*_1_, m3 from *A*_2_ and the rest from (*A*_1_U*A*_2_)^*C*^ corresponding to a high enrichment of signals in *A*_1_ and *A*_2_. Effect sizes of causal variants were sampled from $N(0,\frac{h_{g}^{2}}{m})$. For each simulation, we used 70% of the data to calculate the training summary statistics and randomly divided the rest 30% into two parts for parameter tuning. We also randomly selected half of the training data to calculate summary statistics in order to study the effect of sample size on prediction accuracy.

In order to evaluate the improvement in accuracy, we performed a permutation test to compare the CORs of AnnoPred and LDpred. Suppose the CORs of LDpred and AnnoPred in simulations are *x*_1_, *x*_2_, …, *x*_*n*_ and *y*_1_, *y*_2_, …, *y*_*n*_, respectively. And the hypothesis we want to test is

$$H_{0}:\mu_{x}=\mu_{y}↔H_{1}:\mu_{x}\neq \mu_{y}$$

where *μ*_*x*_ and *μ*_*y*_ represent the population mean of accuracies of LDpred and AnnoPred. We used $\left|\bar{x}-\bar{y}\right|$ as the test statistics and the p value can be calculated as $p=Pr(|\bar{x}-\bar{y})>|{\bar{x}}_{obs}-{\bar{y}}_{obs}||H_{0})$, in which $\bar{x}-\bar{y}$ represents the random variable and ${\bar{x}}_{obs}-{\bar{y}}_{obs}$ represents the actually observed values. We used permutation to approximate the distribution of $\left(\bar{x}-\bar{y}\right)$ when *H*_0_ is true. Specifically, we first pooled *x*_*i*_′*s* and *y*_*i*_′*s* together. Then ${\tilde{x}}_{1},{\tilde{x}}_{2},\ldots ,{\tilde{x}}_{n}$ and ${\tilde{y}}_{1},{\tilde{y}}_{2},\ldots ,{\tilde{y}}_{n}$ were sampled from the pooled data for *N* = 10^6^ times and we calculated $\left(\bar{\tilde{x}}-\overset{-}{\tilde{y}}\right)$ for each ${\tilde{x}}_{i}\prime s$ and ${\tilde{y}}_{i}\prime s$ sampled, which formed the empirical distribution of $\left(\bar{x}-\bar{y}\right)$ under *H*_0_. And the p value could be approximated by $\hat{p}=\frac{\sum_{k=1}^{N}I\{|{\bar{\tilde{x}}}_{k}-{\bar{\tilde{y}}}_{k}|>|{\bar{x}}_{obs}-{\bar{y}}_{obs}|\}}{N}$, in which ${\bar{\tilde{x}}}_{k}-{\bar{\tilde{y}}}_{k}$ represents the sampled test statistic of the kth permutation.

To further study the effect of sample size on prediction performance, we simulated traits using SNPs of chromosome 1, chromosomes 1 and 2, chromosomes 1 to 4 and the whole genome while keeping the same proportion of causal variants and heritability to mimic the situation of increasing sample size. The corresponding relative sample sizes ($N\frac{M}{M_{s}}$, where *N* is the number of individuals, *M* is the total number of variants and *M*_*s*_ is the number of variants used in simulation) for the four scenarios are ~135K, ~67K, 37K and ~11K. For each effective sample size, we simulated traits under four settings: *h*^2^ = 0.25, *p* = 0.001; *h*^2^ = 0.25, *p* = 0.01; *h*^2^ = 0.5, *p* = 0.001; *h*^2^ = 0.5, *p* = 0.01, where *p* represents the proportion of causal variants (**Fig 1**).

## Ethics statement

The study was approved by YALE UNIVERSITY HUMAN INVESTIGATION COMMITTEE with approval number 100 FR1 and 100 FR27.

## Data access

### GWAS summary statistics and validation data

We trained AnnoPred using publicly accessible GWAS summary statistics and evaluated risk prediction performance using individual-level genotype and phenotype data from cohorts independent from the training samples. Only SNPs shared between training and testing datasets were kept in our analyses. Details for each training and testing dataset are provided in **S1 Text** and **S8 Table**.

For Crohn’s disease, we trained the model using summary statistics from International Inflammatory Bowel Disease Genetics Consortium (IIBDGC; N_case_ = 6,333 and N_control_ = 15,056) [25]. Samples from the Wellcome Trust Case Control Consortium (WTCCC) were removed from the meta-analysis and used as the validation dataset (N_case_ = 1,689 and N_control_ = 2,891) [26]. For breast cancer, we trained the model using summary statistics from Genetic Associations and Mechanisms in Oncology (GAME-ON) study (N_case_ = 16,003 and N_control_ = 41,335) [27], and tested the performance using samples from the Cancer Genetic Markers of Susceptibility (CGEMS) study (N_case_ = 966 and N_control_ = 70) [28]. Shared samples between CGEMS and GAME-ON were removed. We used samples from the CIDR-GWAS of breast cancer for trans-ethnic analysis (N_case_ = 1,666 and N_control_ = 2,038) [29]. For rheumatoid arthritis, we used summary statistics from a meta-analysis with 5,539 cases and 20,169 controls to train the model [30]. WTCCC samples were removed from the meta-analysis and used for validation (N_case_ = 1,829 and N_control_ = 2,892) [26]. For type-II diabetes, the training dataset is Diabetes Genetics Replication and Meta-analysis (DIAGRAM) consortium GWAS with 12,171 cases and 56,862 controls [31]. We used samples from Northwestern NUgene Project for validation (N_case_ = 662 and N_control_ = 517) [32]. Samples from Institute for Personalized Medicine (IPM) eMERGE project are used for trans-ethnic analysis (African American: N_case_ = 517 and N_control_ = 213; Hispanic: N_case_ = 477 and N_control_ = 102) [33]. The training dataset for celiac disease is from a GWAS with 4,533 cases and 10,750 controls [34]. Samples in the National Institute of Diabetes and Digestive and Kidney Diseases (NIDDK) celiac disease study were used for validation (N_case_ = 1,716 and N_control_ = 530) [35].

### Software availability

AnnoPred software and source code are freely available online at https://github.com/yiminghu/AnnoPred.

## Supporting information

S1 Fig Enrichment of proportion of cases in the top 5% testing samples with high PRS. (TIFF)

S2 Fig Boxplots of the simulation results in Table 1, p-values of the permutation tests (Methods) quantify the improvement of AnnoPred over PRS without incorporating functional annotations. (TIFF)

S3 Fig Heritability enrichment across GenoCanyon and tissue-specific GenoSkyline annotations. The horizontal line marks no enrichment.
(TIFF)

S4 Fig Per-SNP heritability estimation under constant enrichment in simulation. Dashed line marks the true per-SNP heritability.
(TIFF)

S5 Fig Proportion of SNPs in GenoCanyon and tissue-specific GenoSkyline annotations. (TIFF)

S6 Fig Prediction accuracy of AnnoPred on Crohn’s disease data using different LD radiuses. (TIFF)

S7 Fig Comparing signal strength of SNPs with high priors and low priors in independent validation cohorts with underpowered sample size (<2000). **(A)** Breast cancer **(B)** Type-II diabetes **(C)** Comparing consistency of SNPs’ effect direction between training and testing datasets. Each bar quantifies the proportion of SNPs with consistent effect directions. The association tests and effect size estimation on the testing data are underpowered due to the limited sample size.
(TIFF)

S1 Table GWAS signal enrichment across 61 annotation categories. (XLSX)

S2 Table AUCs of different methods. The highest AUCs are highlighted in boldface.
(XLSX)

S3 Table Comparison of the complete model and AnnoPred with baseline annotations. The highest AUCs are highlighted in boldface.
(XLSX)

S4 Table Comparison of the AnnoPred with method used in (Speed and Balding 2014) for evaluation in real data analysis. The highest AUCs are highlighted in boldface.
(XLSX)

S5 Table Comparison of the AnnoPred with method used in (Speed and Balding 2014) for evaluation in simulation. The highest correlations are highlighted in boldface.
(XLSX)

S6 Table AUCs for trans-ethnic analyses. The highest AUCs are highlighted in boldface.
(XLSX)

S7 Table CORs for trans-ethnic analyses. The highest CORs are highlighted in boldface.
(XLSX)

S8 Table URLs for training and testing datasets. (XLSX)

S9 Table Prediction accuracies after removing SNPs in MHC regions. The highest CORs/AUCs are highlighted in boldface.
(XLSX)

S10 Table Prediction accuracies of AnnoPred when different annotations used. The highest CORs/AUCs are highlighted in boldface.
(XLSX)

S11 Table p-values from the likelihood ratio tests comparing different models. (XLSX)

S12 Table Prediction accuracies on T2D when tuning the parameter in an independent cohort. (XLSX)

S1 Text Details on GWAS summary statistics and validation data. (DOCX)
