# Supplementary figures and images for: Leveraging functional annotations in genetic risk prediction for human complex diseases
Source: PLoS Comput Biol. 2017 Jun 8;13(6):e1005589. doi: 10.1371/journal.pcbi.1005589 (PMC5481142; doi:10.1371/journal.pcbi.1005589)

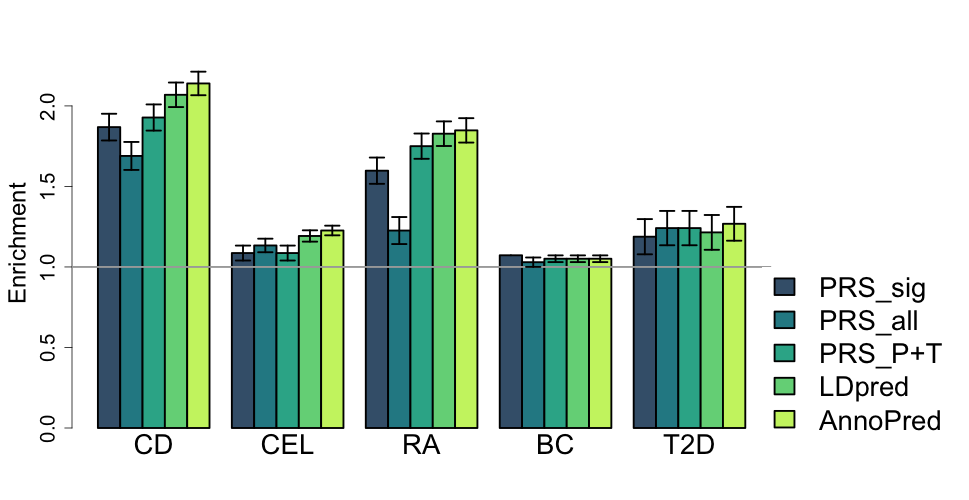

Supplement: S1 Fig — (TIFF) [file pcbi.1005589.s001.tiff]

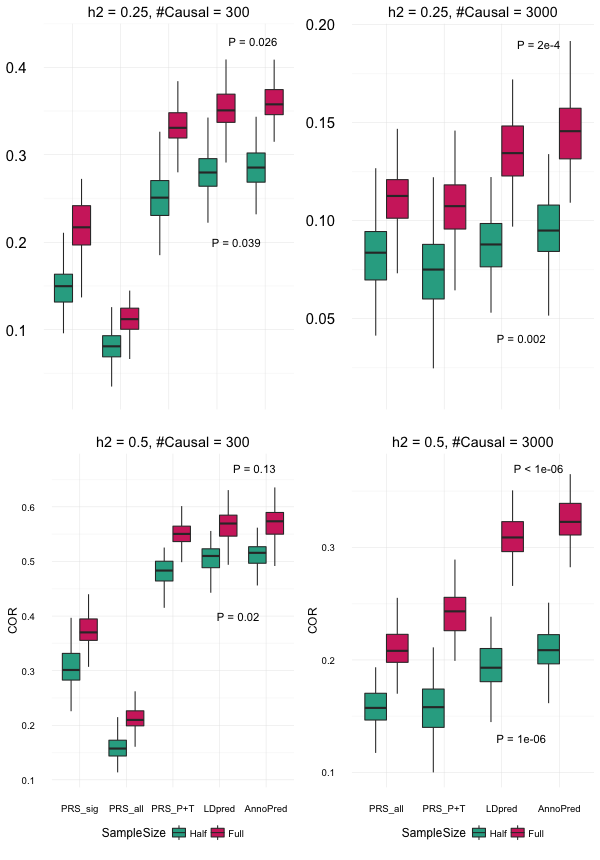

Supplement: S2 Fig — (TIFF) [file pcbi.1005589.s002.tiff]

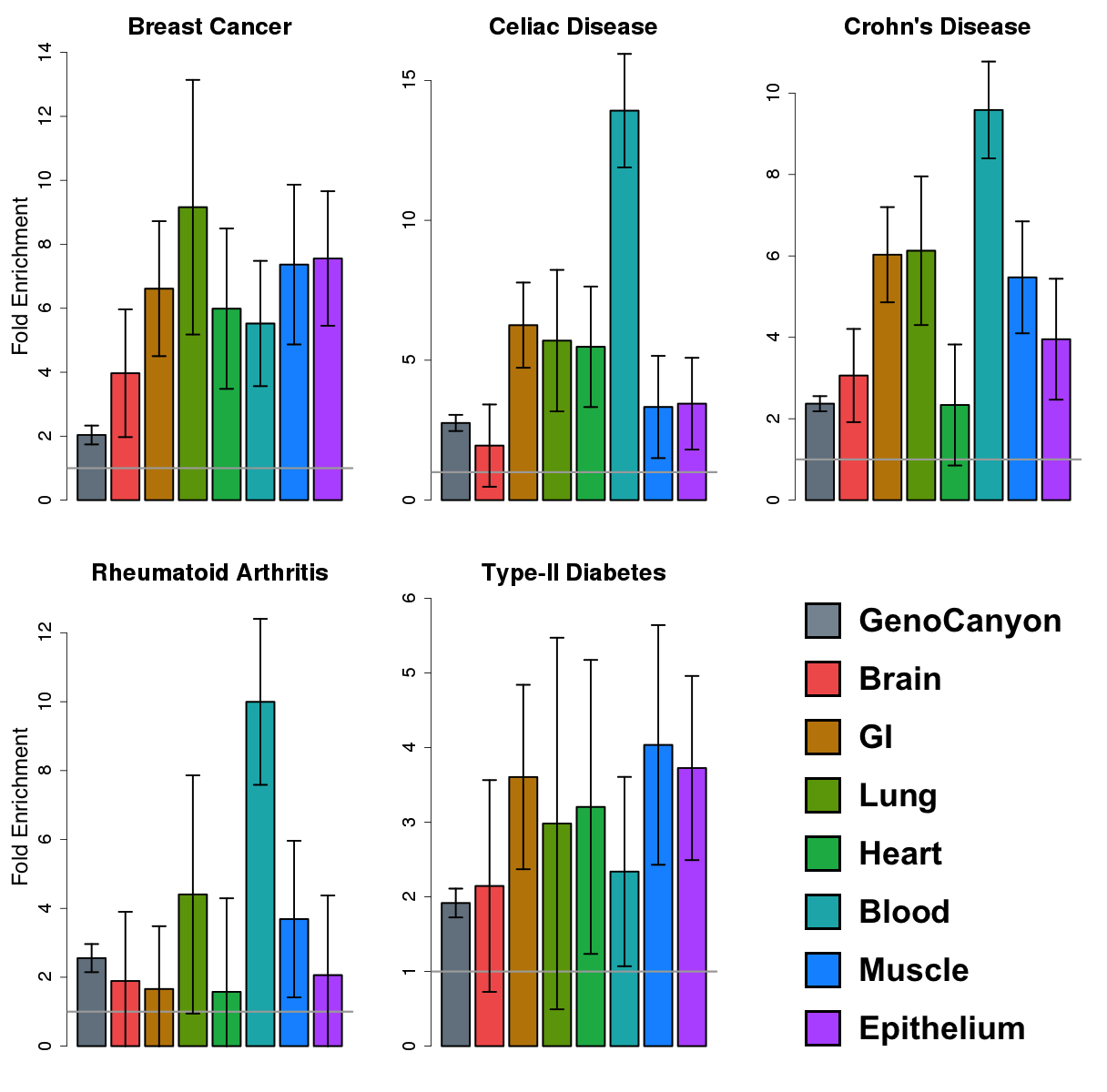

Supplement: S3 Fig — The horizontal line marks no enrichment. (TIFF) [file pcbi.1005589.s003.tiff]

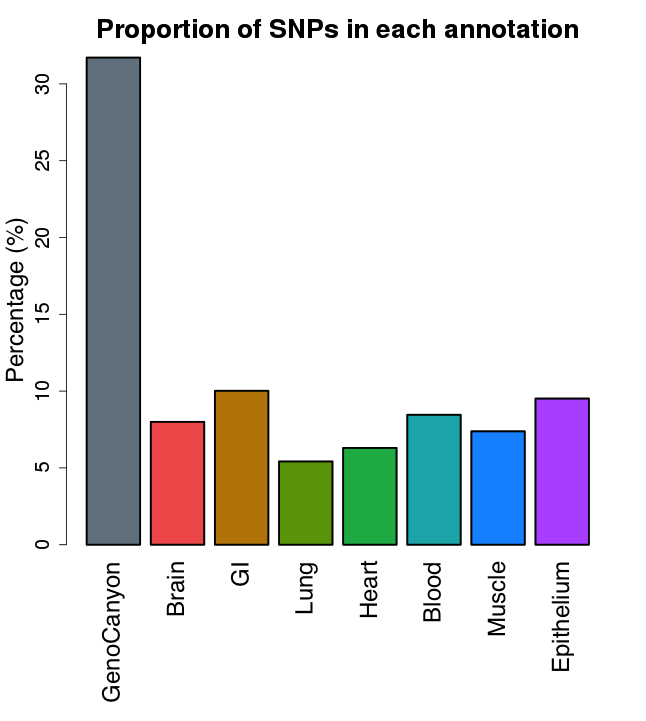

Supplement: S5 Fig — (TIFF) [file pcbi.1005589.s005.tiff]

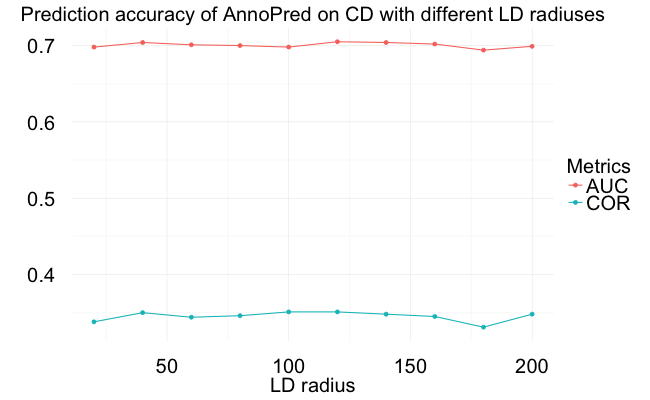

Supplement: S6 Fig — (TIFF) [file pcbi.1005589.s006.tiff]

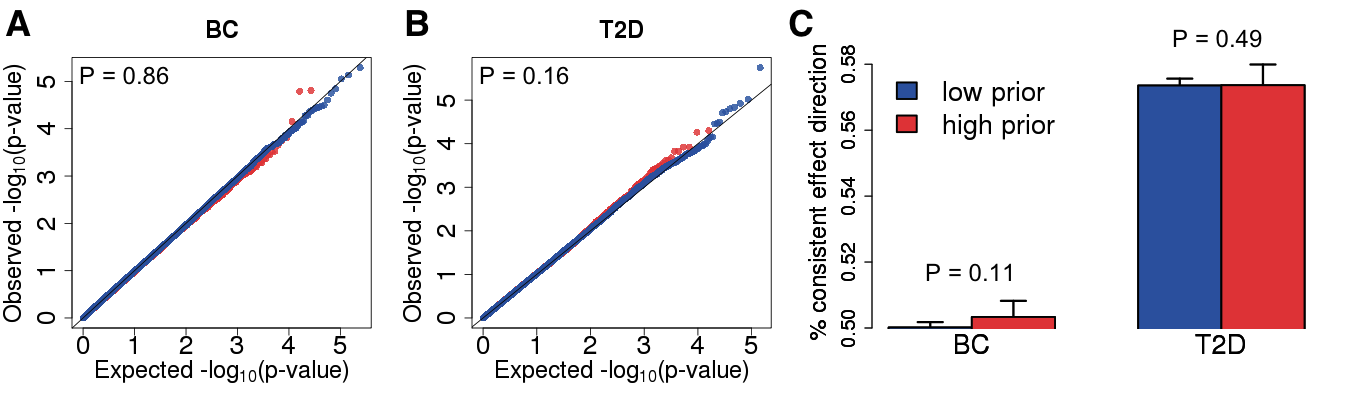

Supplement: S7 Fig — (A) Breast cancer (B) Type-II diabetes (C) Comparing consistency of SNPs’ effect direction between training and testing datasets. Each bar quantifies the proportion of SNPs with consistent effect directions. The association tests and effect size estimation on the testing data are underpowered due to the limited sample size. (TIFF) [file pcbi.1005589.s007.tiff]
