# Supplementary material for: Leveraging functional annotations in genetic risk prediction for human complex diseases
Source: PLoS Comput Biol. 2017 Jun 8;13(6):e1005589. doi: 10.1371/journal.pcbi.1005589 (PMC5481142; doi:10.1371/journal.pcbi.1005589)
Supplement: S1 Text — (DOCX) [file pcbi.1005589.s020.docx]

**Supplemental Text**

**Details on GWAS summary statistics and validation data**

For Crohn’s disease, we used International Inflammatory Bowel Disease Genetics Consortium (IIBDGC) summary statistics (6,333 Crohn’s disease patients and 15,056 controls) [1]. WTCCC was removed from the meta-analysis and used as a validation set [2]. We filtered individuals with genetic relatedness larger than 0.05 and SNPs with a missing rate larger than 1% and a minor allele frequency less than 1%. In addition, we filtered SNPs with ambiguous nucleotides and kept SNPs matched the summary statistics by both rs number and alleles. After QC, the WTCCC cohort consisted of 1,689 cases and 2,891 controls with 218,833 SNPs overlapping the summary statistics.

For breast cancer, we used the Genetic Associations and Mechanisms in Oncology (GAME-ON) summary statistics, consisting of 16,003 cases and 41,335 controls [3]. As for validation data, we first removed individuals overlapped with BPC3 in GAME-ON from Cancer Genetic Markers of Susceptibility (CGEMS) [4]. The validation set consisted of 966 cases and 70 controls with 497,315 SNPs in common. Besides CGEMS, we also used an African-American as validation data to see how the model performs on different population. The data we used is CIDR-GWAS of Breast Cancer in the African Diaspora (CIDR) [5]. After QC, CIDR consisted of 1,666 cases and 2,038 controls with 555,169 SNPs in common.

For rheumatoid arthritis, we used a meta-analysis consisting of 5,539 cases and 20,169 controls [6]. WTCCC was removed from the meta-analysis and used as a validation set [2]. After QC, WTCCC cohort consisted of 1,829 cases and 2,892 controls with 274,486 SNPs in common.

For type-II diabetes, we used Diabetes Genetics Replication and Meta-analysis (DIAGRAM) consortium GWAS summary statistics with 12,171 cases and 56,862 controls [7]. For testing data, we used Northwestern NUgene Project and after QC it consisted of 662 cases and 517 controls with 479,345 SNPs in common [8].

For celiac disease, we used a GWAS consisting of 4,533 cases and 10,750 controls [9]. The National Institute of Diabetes and Digestive and Kidney Diseases (NIDDK) celiac disease data was used as validation data [10]. After QC, it consisted of 1,716 cases and 530 controls with 504,785 SNPs in common.

**References**

1. Franke A, McGovern DP, Barrett JC, Wang K, Radford-Smith GL, Ahmad T, et al. Genome-wide meta-analysis increases to 71 the number of confirmed Crohn's disease susceptibility loci. Nat Genet. 2010;42(12):1118-25. doi: 10.1038/ng.717. PubMed PMID: 21102463; PubMed Central PMCID: PMCPMC3299551.

2. Burton PR, Clayton DG, Cardon LR, Craddock N, Deloukas P, Duncanson A, et al. Genome-wide association study of 14,000 cases of seven common diseases and 3,000 shared controls. Nature. 2007;447(7145):661-78.

3. Michailidou K, Hall P, Gonzalez-Neira A, Ghoussaini M, Dennis J, Milne RL, et al. Large-scale genotyping identifies 41 new loci associated with breast cancer risk. Nature genetics. 2013;45(4):353-61.

4. Hunter DJ, Kraft P, Jacobs KB, Cox DG, Yeager M, Hankinson SE, et al. A genome-wide association study identifies alleles in FGFR2 associated with risk of sporadic postmenopausal breast cancer. Nature genetics. 2007;39(7):870-4.

5. Zheng Y, Ogundiran TO, Falusi AG, Nathanson KL, John EM, Hennis AJ, et al. Fine mapping of breast cancer genome-wide association studies loci in women of African ancestry identifies novel susceptibility markers. Carcinogenesis. 2013:bgt090.

6. Stahl EA, Raychaudhuri S, Remmers EF, Xie G, Eyre S, Thomson BP, et al. Genome-wide association study meta-analysis identifies seven new rheumatoid arthritis risk loci. Nature genetics. 2010;42(6):508-14.

7. Morris AP, Voight BF, Teslovich TM, Ferreira T, Segre AV, Steinthorsdottir V, et al. Large-scale association analysis provides insights into the genetic architecture and pathophysiology of type 2 diabetes. Nature genetics. 2012;44(9):981.

8. McCarty CA, Chisholm RL, Chute CG, Kullo IJ, Jarvik GP, Larson EB, et al. The eMERGE Network: a consortium of biorepositories linked to electronic medical records data for conducting genomic studies. BMC medical genomics. 2011;4(1):13.

9. Dubois PC, Trynka G, Franke L, Hunt KA, Romanos J, Curtotti A, et al. Multiple common variants for celiac disease influencing immune gene expression. Nature genetics. 2010;42(4):295-302.

10. Garner C, Ahn R, Ding YC, Steele L, Stoven S, Green PH, et al. Genome-wide association study of celiac disease in North America confirms FRMD4B as new celiac locus. PLoS One. 2014;9(7):e101428. Epub 2014/07/08. doi: 10.1371/journal.pone.0101428. PubMed PMID: 24999842; PubMed Central PMCID: PMCPmc4084811.
